# Polyphenols from halophytes and modified atmosphere packaging improve sensorial and biochemical markers of quality of common dolphinfish (*Coryphaena hippurus*) fillets

**DOI:** 10.1002/fsn3.337

**Published:** 2016-03-30

**Authors:** Concetta Maria Messina, Gioacchino Bono, Rosaria Arena, Mariano Randazzo, Simona Manuguerra, Andrea Santulli

**Affiliations:** ^1^ Dipartimento di Scienze della terra e del Mare DiSTeMUniversità degli Studi di PalermoLaboratorio di Biochimica Marina ed Ecotossicologia Via G. Barlotta 4Trapani91100Italy; ^2^ Istituto per l'Ambiente Marino CostieroConsiglio Nazionale delle RicercheVia L. Vaccara 61Mazara del Vallo91026Italy; ^3^ Istituto di Biologia marinaConsorzio Universitario della Provincia di TrapaniVia G. Barlotta 4Trapani91100Italy

**Keywords:** Fish, modified atmosphere packaging, polyphenols, shelf‐life

## Abstract

Quality and shelf‐life of whole and filleted *Coryphaena hippurus*, stored with modified atmosphere packaging (MAP) and natural antioxidants from halophytes (HAL), were investigated. Fillets were divided into control, simply sealed in trays; MAP, preserved by modified atmosphere (45% CO_2_, 50% N_2_, 5% O_2_); and MAP‐HAL, pretreated with antioxidants and preserved by MAP. Whole and filleted fish were stored at −1 ± 0.5°C for 18 days. The quality of the samples was analyzed at the time of packaging and after 3, 6, 9, 12, 15, and 18 days. The MAP and MAP‐HAL groups maintained the best sensorial profile, pH, and drip loss with respect to the untreated fillets. Higher levels of total volatile basic nitrogen (TVB‐N) and oxidized proteins were observed in untreated samples with respect to the MAP and MAP‐HAL groups. The principle component analysis revealed a different quality profile for untreated and MAP‐treated fish.

## Introduction

Recently, consumers began associating the consumption of seafood with beneficial effects on human health, which has led to an increasing demand for seafood. In order to meet these demands of both domestic and public consumption, new fish products and new technologies, able to extend the shelf‐life of seafood, are of interest. Currently, many new fish and fishery products are imported from aquaculture in Pacific areas to the European market as deep‐frozen fillets, not always of good quality (Karl et al. 2014).

The growing demand for seafood can be met by introducing new fish products, by promoting the consumption of local resources and low commercial value fish species, and by adopting innovative methods of processing, which are able to extend the shelf‐life of fish. Modified atmosphere packaging (MAP) has received increasing attention, also in the seafood industry. MAP is defined as the enclosure of food products in gas‐barrier materials; the gaseous environment inside the package is changed in order to reduce spoilage and extend the shelf‐life of the food product (Sivertsvik et al. 2002; Masniyom 2011). Additionally, many recent studies have focused on the use of natural polyphenols, instead of synthetic products, for extending the shelf‐life of products in the seafood industry (Mastromatteo et al. 2010).

We designed the present study to investigate the effect of coupling MAP with natural antioxidant treatment, on biochemical quality traits of dolphinfish (*Coryphaena hippurus*), a Mediterranean fisheries resource. Results from another recent study demonstrated that MAP, coupled with natural antioxidants, prevented the oxidation of polyunsaturated fatty acids and the production of molecules that lead to peroxidation, preserving the quality of dolphinfish (Messina et al. 2015). In this study, we aimed to verify the effects of the same packaging process on others aspect of quality, related to the evolution of the protein composition of dolphinfish fillets. In fact, during fish storage, chemical and enzymatic changes affect proteins and ultimately give rise to off‐flavors, odors, and amino acid breakdown products (Antoine et al. 2002a). Shelf‐life evolution of fishes is known to be species‐specific and related to the chemical composition of the fish (Antoine et al. 2002a). Therefore, the observations of quality by an integrated sensorial and biochemical approach can help to describe the susceptibility of a species to spoilage and to identify the most effective method of processing. As source of antioxidants, in this study, we used a species of halophyte, *Halocnemum strobilaceum*, which is endemic in the region of Sicily, where the dolphinfish were caught. This halophyte, as previously reported, is rich in polyphenols, tested for antioxidant power and total polyphenol content (Messina et al. 2015). In addition, in this study, we compared data of filleted fish to whole fish, stored at the same temperature of fillets.

## Materials and Methods

### Fish sampling and processing

Fish were caught from the west side of the Strait of Sicily in the central Mediterranean Sea in October 2014. Approximately 60 fish (average total length, 50.2 ± 2.78 cm; average total weight, 980.2 ± 123.45 g) were caught in the same haul. The fish were washed in flowing seawater, put on ice and delivered to the laboratory in less than 1 h. Upon arrival at the laboratory, 12 fish were maintained to be preserved on the whole (WH) and 40 fish were washed under running tap water, headed, gutted, cleaned, and rewashed prior to be filleted.

From the 80 obtained fillets, eight were utilized for the analyses at T zero and the others (72) were destined to the shelf‐life experiments. The 72 fillets were randomly divided into three lots that were preserved in three different ways: 24 fillets of the control group (CO) were simply sealed in 12 boxes (two fillets/box); 24 fillets of the MAP group were preserved in 12 boxes (two fillets/box) under modified atmosphere (45% CO_2_, 50% N_2_, 5% O_2_); and 24 fillets of the MAP‐HAL group were immersed in a solution of natural antioxidants from halophytes and afterward preserved in 12 boxes (2 fillets/box), with the same modified atmosphere conditions adopted for the MAP group. The treatment with the antioxidant solution was done according to Messina et al. (2015) by immersing the fillets in 1 L of antioxidant solution (10 g L^−1^ of distilled water) for 2 min.

### Packaging materials and gas composition

The gas blends were prepared using a gas mixer (MAP Mix 9000, PBI‐Dansensor A/S, Ringsted, Denmark). All samples (2 fillets/package) were packaged in fully transparent APET/EVOH/PE barrier boxes (bag volume: 1800 cc; laminate density: 1.39 g/cm^3^; thickness: 500 *μ*m) with an oxygen permeability (at 23°C and 0% relative humidity [RH]) of 1.8 cm^3^ m^−2^ day^−1^ atm^−1^ and water vapor permeability of 4 g m^−2^ day ^−1^. To prevent the undesirable accumulation of tissue fluids, an absorbent food pad (LLP34, Cryovac‐Sealed Air, NJ) was inserted between the fish and the bottom of the packaging bag. The bags were heat sealed with a barrier shrink film that was 25‐*μ*m thick (LID 2050, Cryovac‐Sealed Air) with O_2_ and CO_2_ permeabilities (at 23°C and 0% RH) of 24 and 95 cm^3^ m^−2^ day^−1^ atm^−1^, respectively. The heat‐sealing operation was completed using a semiautomatic packaging machine (Mondini, Brescia, Italy). A gas‐to‐product ratio of 2.5:1 was used for samples packed under MAP.

### Storage and sampling

Both fillets and WH fish were stored in a refrigerator at −1 ± 0.5°C for 18 days. We analyzed the quality of the samples by sensorial, chemical, and biochemical methods at the time of packaging and after 3, 6, 9, 12, 15, and 18 days of storage. We analyzed two WH fish and two boxes, each containing two fillets, from each treatment group on each day of sampling.

### Sensorial analyses

Seven members of the laboratory, trained in fish quality evaluation, completed the sensory evaluations of the WH fish and fillets preserved under the experimental conditions (CO, MAP, and MAP‐HAL), according to the scheme adopted from Antoine et al. (2002b) for dolphinfish, independently, under the same environmental condition and after the box were opened. The panelists assessed the attributes of odor (very fresh, slightly fishy, moderately fishy, and ammoniacal), color (very bright, bright, dull, and brown), gaping (none, slight, moderate, and excessive), and texture (very firm, firm, soft, and very soft) (Antoine et al. 2002a). A categorical scale of 1–10 was used for each attribute; a score of 1–2 indicated very poor quality and a score of 9–10 indicated excellent quality. Sensory evaluations were completed at the same time on each day of evaluation. The results are expressed as the mean score assigned by the panelists.

### Chemical and physical analyses: pH and drip loss

Determinations of pH and drip loss were completed according to the method of Benjakul et al. (1997). The samples were homogenized by ultraturrax T25 (KA®‐Werke GmbH & Co. KG, Staufen, Germany) with 10 volumes of deionized water (w/v) at a speed of 11,000 rpm for 1 min. The pH of the homogenate was measured using a microprocessor controlled pH meter (CRISON MicropH 2001, Barcelona, Spain). The drip loss was measured gravimetrically only in filleted fish, after 48 h of packaging (day 3 of storage). First, the entire package (sample and film) was weighed. Then, the sample and any purge were removed from the package, and the fish and the entire package surface were wiped clean with a paper towel. Finally, the fish sample was placed back into its package and reweighed. The drip's mass (g) was divided by the initial mass of the product (g) and reported as a percentage (%).

### Biochemical analyses

Fillets from treatments and from WH fish were homogenized and used for analyses, in triplicate, to assess the quality and shelf‐life of the fish. Aliquots of the homogenate were processed for moisture (method 950.46), ash (method 938.08), and crude protein (method 981.10), according to the AOAC (1990) procedures, and total lipid (Folch et al. 1957) contents.

The total volatile basic nitrogen (TVB‐N) content was determined by direct distillation of the homogenized samples, according to the EU Commission Decision 95/149/EC (EEC, 1995). Protein deterioration by oxidation was determined according to the method described by Lin and Lin (2005). Each 0.4 mL aliquot of the homogenate was mixed thoroughly with 4 mL of distilled water and 5 mL chloroform–methanol (2:1, v/v); the total sample was centrifuged at 400 rpm for 20 min. The water layer was collected and used to determine the fluorescence intensity (excitation wavelength of 340 nm and emission wavelength of 445 nm) using a spectrofluorometer (Cary Eclipse Fluorescence Spectrophotometer, Agilent Technologies, CA).

The molecular weights of the proteins in the fish samples were determined with sodium dodecyl sulfate polyacrylamide gel electrophoresis (SDS‐PAGE). Total proteins (TP) were extracted from the fillet homogenate (1:2 w/v) by a solution of HEPES 10 mmol/L (pH 7.4), NaCl 4 mol/L, EDTA 2 mmol/L, EGTA 2 mmol/L, and a cocktail of protease inhibitors. TP were diluted to a final protein concentration of 6 mg/mL in a sample buffer (2 mL of 2‐mercaptoethanol, 20 mL 10% SDS, and 5 mL 0.05% bromophenol blue) in 10 mmol/L Tris/HCl and 1 mmol/L EDTA at pH 8.0. The final volume was adjusted to 100 *μ*L. The samples were heated for 10 min in a boiling water bath and centrifuged at 16,000*g* for 1 min. The electrophoresis was performed on a Bio‐Rad system using a 4–15% Mini‐PROTEAN polyacrylamide gel gradient (Bio‐Rad Laboratories, Inc., CA). The gel was run at 120 V and 20 mA at 15°C. The gel was stained with Coomassie blue (0.1% PhastGel Blue R solution in 30% methanol and 10% acetic acid), destained with 30% methanol and 10% acetic acid, and preserved in 10% acetic acid and 5% glycerol. The approximate molecular weights of the proteins were determined using wide range molecular weight standards (PrecisionPlus standards, Bio‐Rad).

### Statistical analyses

The statistical differences were evaluated for each parameter by the analysis of variance (ANOVA). The differences among the mean values were subjected to the Student–Newman–Keuls test (SNK). Before analysis, the degree of heterogeneity was assessed by the Cochran's test (Underwood 1997). Data were processed for one‐way ANOVA and principal component analysis (PCA) by Statistica (version 8.0, Statsoft, Inc., Oklahoma).

## Results and Discussion

Fish packed as ready‐to‐use products could respond to the growing demand for minimally processed food and combine high quality with high convenience (Torrieri et al. 2011). MAP, combined with molecules of natural origin, environmentally safe, inexpensive, and not limiting is one of the most successful preservation techniques available to extend the shelf‐life of seafood (Mastromatteo et al. 2010). This technique is especially desirable for underutilized fisheries species in order to increase the market of less‐popular species and diversify the offerings of commercial seafood. Several reports are available on dolphinfish fillet quality, including studies of fillet refrigeration (Du et al. 2000; Antoine et al. 2002a,b) and fillets processed by high pressure and cold‐smoking techniques (Yagiz et al. 2007; Gómez‐Guillén et al. 2009; Gómez‐Estaca et al. 2013). There is increasing interest in expanding the use of this resource by applying alternative methods of processing for preservation and storage.

### Sensorial analyses

Consumers use postmortem biological changes, including smell and other sensory attributes, to assess fish quality. As such, they assess some characteristics not usually considered by experts. Fish tissues contain large quantities of nitrogenous compounds that generate volatile compounds responsible for the variation in some sensorial attributes, such as odor, which are generally perceived to indicate putrefaction (Antoine et al. 2002a). Of these malodorous compounds, volatile bases are considered the most characteristic feature of fish spoilage (Shewan et al. 1971).

**Figure 1:**
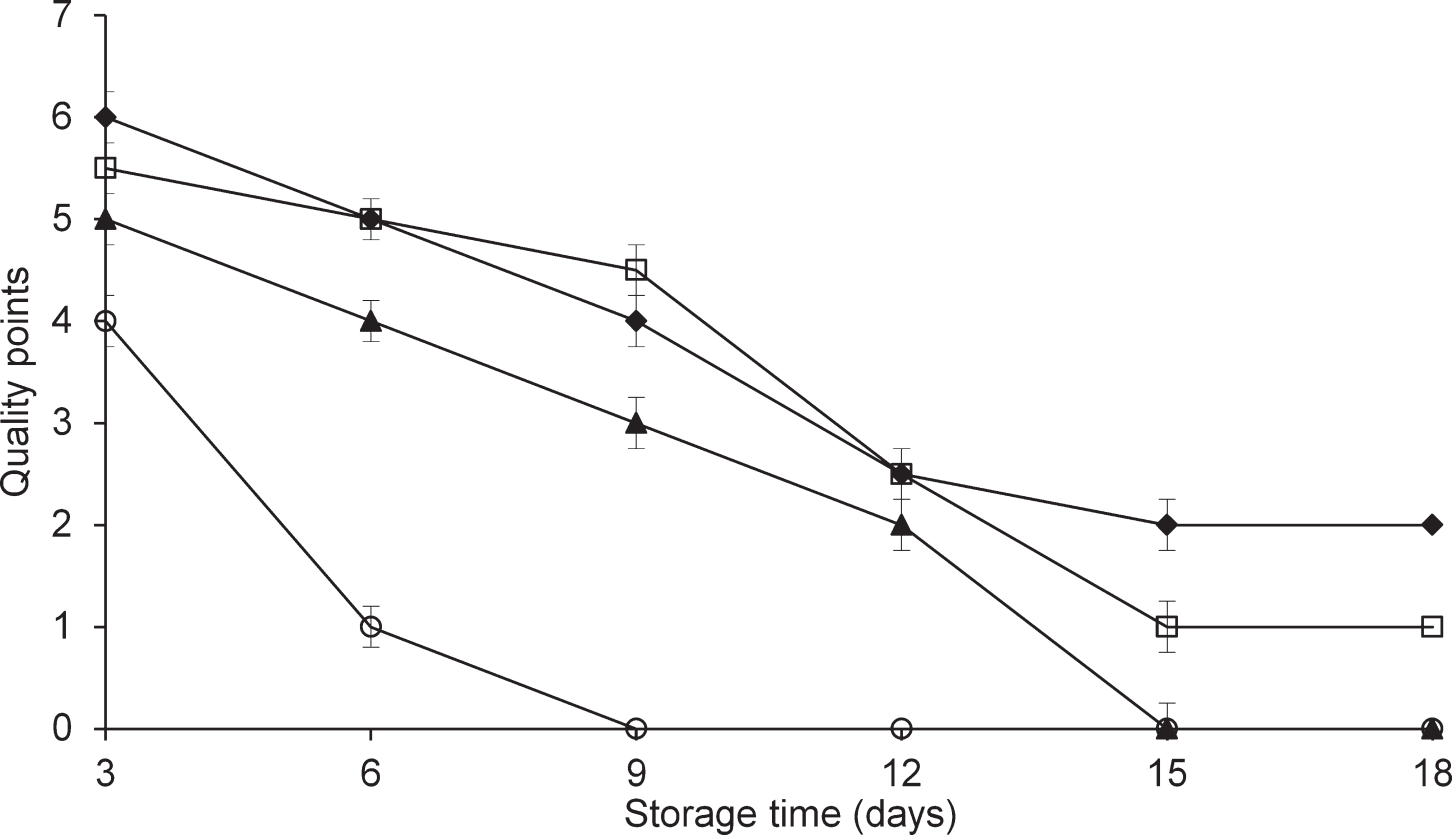
Sensorial evaluation scores attributed to *Coryphaena hippurus* from 3 to 18 days of storage at −1°C according to the method of preservation: untreated control group (CO) (▲), modified atmosphere packaging MAP (□), modified atmosphere packaging combined with natural antioxidant treatment MAP‐HAL (halophytes) (♦), whole fish (WH) (○). Error bar are smaller than symbols. Each point represents the mean of seven independent determinations with a standard deviation smaller than 0.4.

The results of the sensory evaluations of WH and filleted fish are presented in Figure 1.

At the time of arrival in the laboratory, fish were in the extra freshness status, represented by a score of 10, referred from all panelists. The sensorial evaluation of WH and filleted fish, from the third day onward, is represented in Figure 1. WH fish, at day 6 of storage, were considered not acceptable for consumption. In general, samples stored under MAP and MAP‐HAL showed better appearances than samples stored under CO conditions, and the MAP and MAP‐HAL groups maintained a level of acceptability until day 9. After day 9, fillets preserved with MAP‐HAL maintained the best general sensorial characteristics, followed by the MAP group (*P* < 0.05; Fig. 1). Results of the sensorial evaluations for WH fish agree with data reported by Antoine et al. (2002b) on fillets of the same species, which were preserved at 7°C, in which significant variations in sensorial traits start on day 3 of storage.

The combination of MAP with antioxidants (MAP‐HAL), resulted in a better maintenance of fish sensorial quality, which is similar to findings obtained with other fish species processed in a similar manner, such as cod (Wang et al. 2008), seabream, and salmon fillets (Giménez et al. 2004, 2005). Our results confirm that MAP, in combination with refrigeration at low temperatures and treatment with natural antioxidants, can maintain some sensorial aspects of fish quality, which are the first parameters evaluated by consumers in food grading.

### Chemical and physical analyses: pH and drip loss

**Figure 2:**
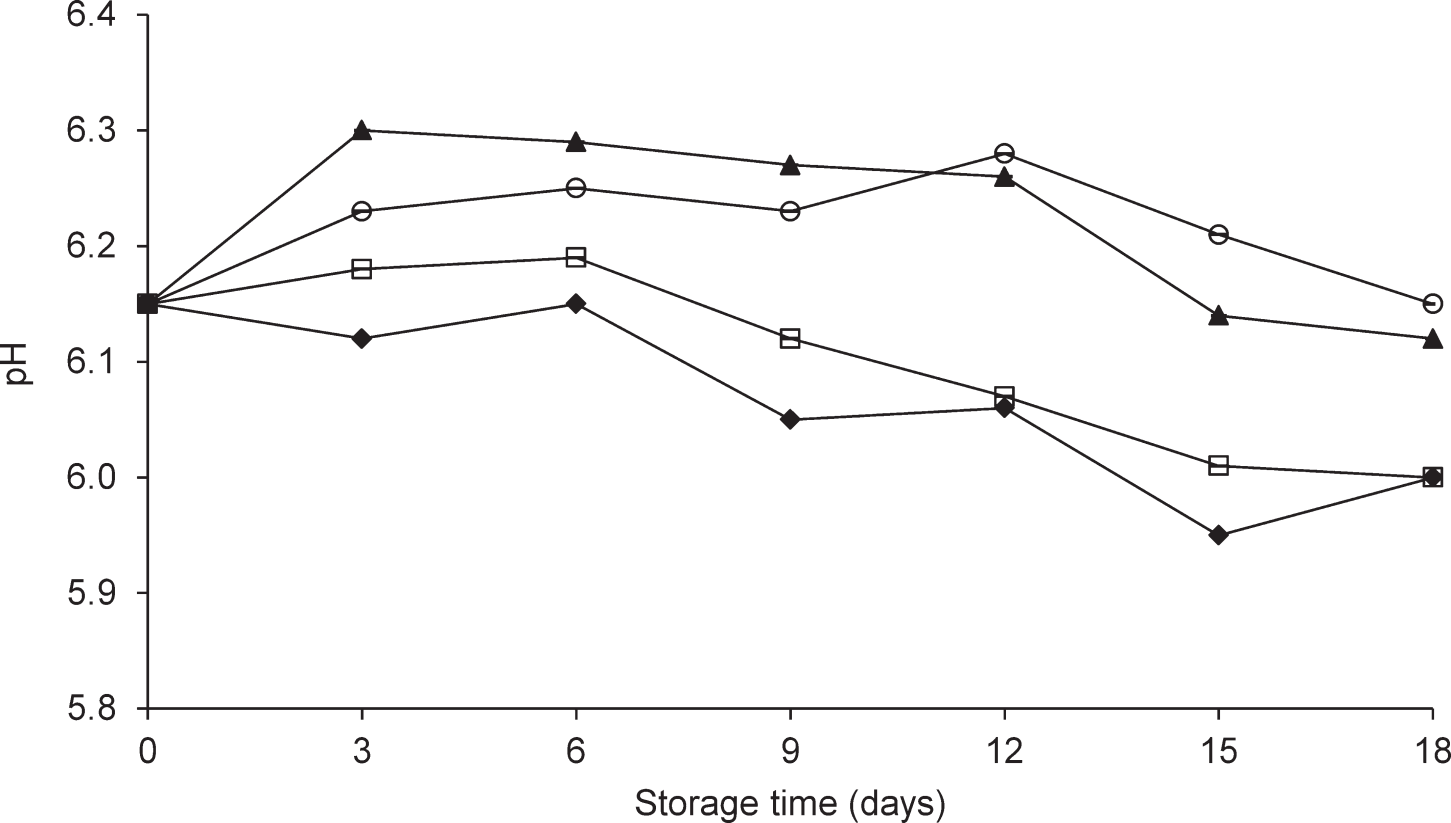
pH variations in *Coryphaena hippurus* during 18 days of storage at −1°C according to the method of preservation: untreated control group (CO) (▲), modified atmosphere packaging MAP (□), modified atmosphere packaging combined with natural antioxidant treatment MAP‐HAL (halophytes) (♦), whole fish (WH) (○). Each point represents the mean of four independent determinations with a standard deviation smaller than 0.5.

The increase in pH during fish storage is commonly associated with spoilage and is considered a general consequence of the accumulation of basic amines in the muscle (Torrieri et al. 2006).The pH values registered during the storage of WH fish and fillets are shown in Figure 2.

Modified atmosphere packaging (MAP)‐HAL and MAP fillets tended to have lower pH values than WH and CO samples during the entire storage period (*P* < 0.05). The pH increase in untreated samples can be explained by the production of basic amines, which are responsible for rapid spoilage (Ordóñez et al. 2000; Torrieri et al. 2006; Kostaki et al. 2009).

The lower pH for samples stored under MAP can be attributed to the effect of CO_2_ in inhibiting protein breakdown and, thus, amine production. Similar trends were observed in other studies of fish processed by MAP (Ordóñez et al. 2000; Cakli et al. 2006; Torrieri et al. 2006; Goulas and Kontominas 2007; Kostaki et al. 2009).

**Figure 3:**
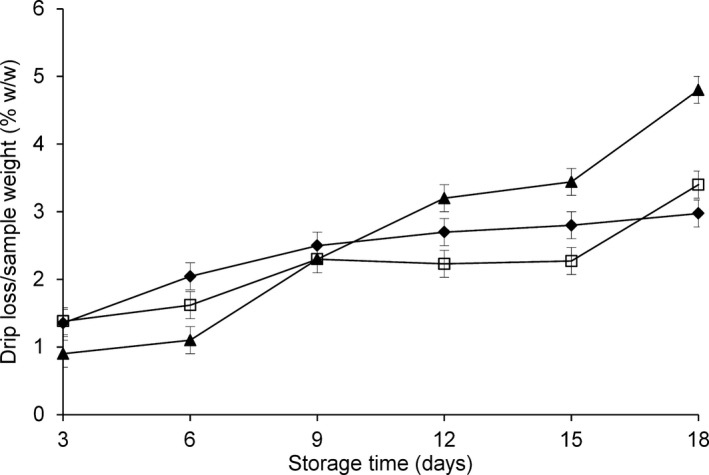
Drip loss variations (%w/w) in fillets of *Coryphaena hippurus* during 18 days of storage at −1°C according to the method of preservation: untreated control group (CO) (▲), modified atmosphere packaging MAP (□), modified atmosphere packaging combined with natural antioxidant treatment MAP‐HAL (halophytes) (♦).

The pH variation modifies protein quality and properties and influences water‐holding capacity, which is responsible for the drip loss that is negatively associated with fish quality evolution. In the present study, drip loss increased slightly with storage time in all fillet samples (Fig. 3). No obvious differences in drip loss were observed among the treatment groups during the first 9 days of storage, which confirms the favorable response of this species to filleting and water‐holding capability. Drip loss was higher in the CO group during the latter part of the storage period (Fig. 3).

The drip loss reached approximately 2% in the MAP group, which was lower than the values (4–8%) reported for MAP‐preserved cod (Dalgaard et al., ), salmon (Sivertsvik et al. 2002), sea bream (Goulas and Kontominas 2007), and sea bass (Torrieri et al. 2006); other studies reported that super‐chilled MAP storage did not lead to excessive drip formation and that water losses lower than 3–5% do not significantly affect the juiciness of fish flesh (Torrieri et al. 2006). The inclusion of absorbing pads in the packages, as in our study, can mitigate the negative impact of drip loss on both quality and consumer acceptability (Torrieri et al. 2006).

### Biochemical analyses

**Figure 4:**
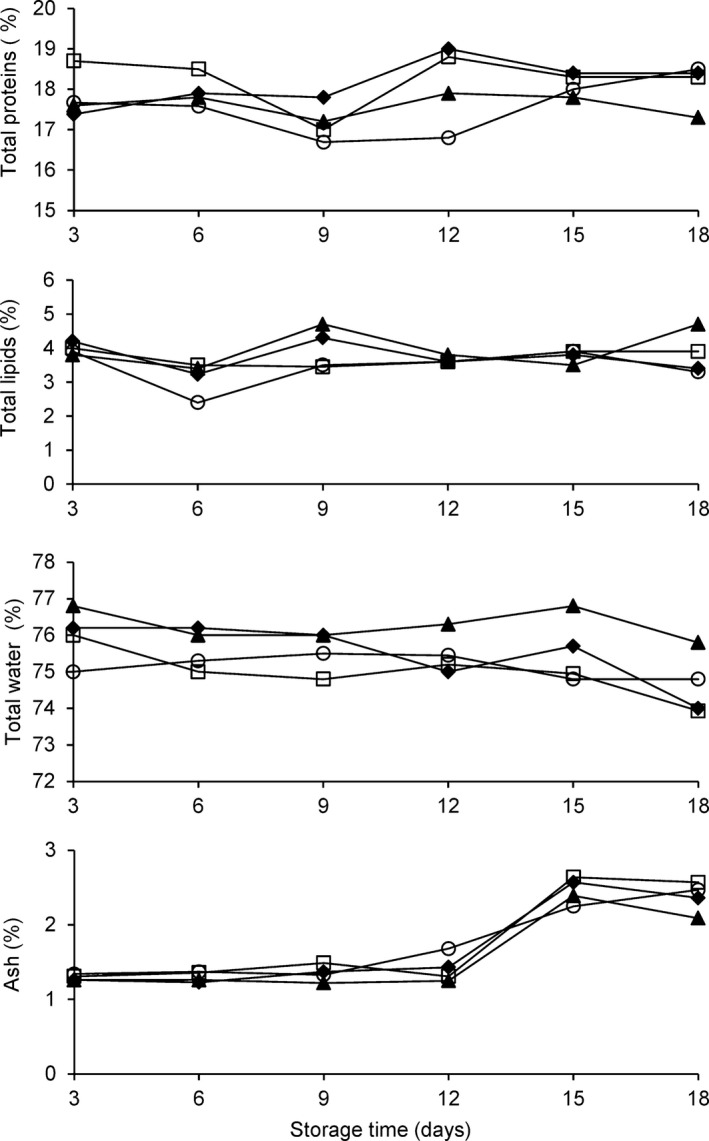
Proximate composition (%) of fillets of *Coryphaena hippurus* during 18 days of storage at −1°C according to the method of preservation: untreated control group (CO) (▲), modified atmosphere packaging MAP (□), modified atmosphere packaging combined with natural antioxidant treatment MAP‐HAL (halophytes) (♦), whole fish (WH) (○). Each point represents the mean of four independent determinations with a standard deviation smaller than 0.4.

The proximate compositions of WH dolphinfish and fillets, preserved in three different manners, are shown in Figure 4. In general, the proximate composition, determined on fresh fish at t zero, agree with data reported by Yagiz et al. (2005, 2007). The total proteins account for 18.97 ± 0.15%, total lipids 4.6 ± 0.62%, total water 72.68 ± 0.29%, and ash 1.47 ± 0.3%. The evolution of proximate composition of whole fish and fillets, after 3 days, is represented in Figure 4.

Modified atmosphere packaging and MAP‐HAL fillets did not show significant variations in composition during the storage time as did WH and CO samples (Fig. 4). This observation confirms that MAP, with or without natural extracts, can contribute to maintain the global quality of nutritional components in this species and that could be an alternative to synthetic preservatives used in the food industry.

Only a small reduction in water content (approximately 3–5%) was observed in fillets from the MAP and MAP‐HAL groups during the entire storage period and it was responsible of the slight protein content variation during the preservation (Fig. 4). A similar trend was observed in sea bass preserved under MAP and can be considered a result of species‐specific characteristics and CO_2_ concentration (Torrieri et al. 2006). In fact, the increase in CO_2_ concentration enhances exudation by acidifying fish muscle and reduces the water‐holding capacity of fish proteins (Pastoriza et al. 1998).

**Figure 5:**
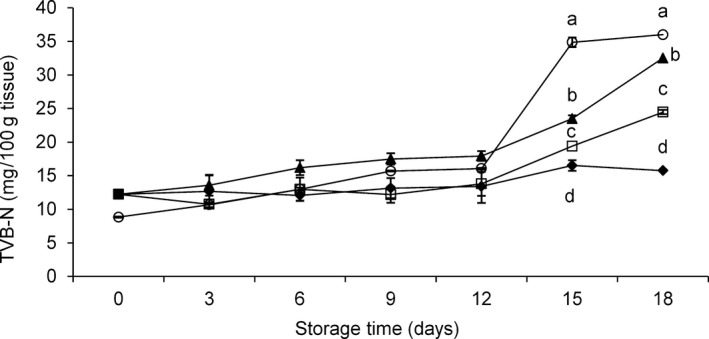
TVB‐N variations (mg/100 g tissue) in fillets of *Coryphaena hippurus* during 18 days of storage at −1°C according to the method of preservation: untreated control group (CO) (▲), modified atmosphere packaging MAP (**□**), modified atmosphere packaging combined with natural antioxidant treatment MAP‐HAL (halophytes) (♦), whole fish (WH) (○). Different superscript letters, within the same storage time, indicate significant differences among treatments (a, b, c: *P* < 0.05).

Many indicators are available as biomarkers of fish spoilage, especially nitrogen degradation, including TVB‐N, biogenic amines, ammonia, however none of these markers are univocally applicable. TVB‐N is universally considered an easy and reliable indicator of fish shelf‐life if coupled with others indicators, and its correlations with other biomarkers of shelf‐life have been demonstrated for dolphinfish (Antoine et al. 2002b). The TVB‐N levels recorded in the WH fish and fillets in our study are presented in Figure 5. Until day 12, neither unprocessed nor processed samples showed a significant increase in TVB‐N levels, although fillets in the MAP and MAP‐HAL groups had lower TVB‐N values than fillets in the CO group (Fig. 5). After day 12, only WH fish reached the threshold of spoilage (30 mg of TVB‐N/100 g), followed by samples of CO group. Fillets in the MAP‐HAL and MAP groups showed TVBN values below the threshold for spoilage, even after 15 days of storage (Fig. 5).

The levels of TVB‐N did not differ between fillets of the MAP and MAP‐HAL group, until day 12; after day 12, MAP samples exhibited an increase in TVB‐N production, with respect to MAP‐HAL (*P* < 0.05). The inhibitory effect of CO_2_ on the production of TVB‐N was more pronounced in samples from the MAP‐HAL group, suggesting a synergistic role of MAP and antioxidants (Fig. 5).

For all samples in our study, the levels of TVB‐N were lower than those recorded in the same species and stored under refrigeration at 7°C (Antoine et al. 2002b); our results were similar to those recorded in cold‐smoked dolphinfish, which supports the fact that some processing techniques, coupled with natural antioxidants, can significantly contribute to the preservation of quality and the extension of shelf‐life of seafood by limiting the production of TVB‐N (Gómez‐Guillén et al. 2009). The validity of this indicator in evaluating the shelf‐life of dolphinfish was established by Antoine et al. (2002b), who also reported a positive correlation between TVB‐N levels and sensorial evaluations.

Other fish species processed by coupling MAP with antioxidants exhibited a decrease in TVB‐N levels, which confirms the effectiveness of this processing method for preserving the quality of fish (Kostaki et al. 2009; Mastromatteo et al. 2010). The European Union (Commission Decision 1995–95/149/EEC) set an upper limit of 25–35 mg TVB‐N per 100 g of fish for consumption, but additional data are needed to provide new limits for processed fish, as suggested by Gómez‐Guillén et al. (2009).

Others aspects of protein deterioration can be considered in the evaluation of the shelf‐life of fish, such as the loss of quality, due to protein oxidation. Protein oxidation is one of the factors influencing the deterioration of fish meat, although most enzyme activity is slowed or inhibited during frozen storage. Srinivasan and Hultin (1994) reported that protein denaturation was mainly affected by lipid oxidation, suggesting that protein oxidation is likely coupled with lipid oxidation.

**Figure 6:**
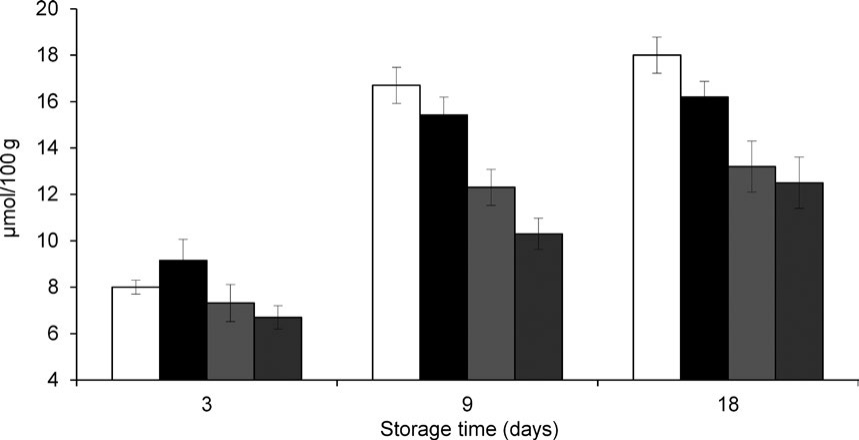
Protein degradation (*μ*mol/g) in fillets of *Coryphaena hippurus* during 18 days of storage at −1°C according to the method of preservation: untreated control group (CO) (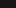), modified atmosphere packaging MAP (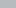), modified atmosphere packaging combined with natural antioxidant treatment MAP‐HAL (halophytes) (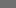), whole fish WH (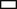).

Our previous results showed that storage with MAP and natural antioxidants can reduce lipid peroxidation in dolphinfish (Messina et al. 2015). In this study, the analysis of protein degradation by oxidation revealed less oxidation than that reported by Lin and Lin (2005) in both untreated and treated samples of bonito fillets (Fig. 6). Furthermore, higher levels of oxidized proteins were observed in WH fish and CO fillets than in MAP and MAP‐HAL fillets. These findings confirm that the fillets resulted protected against oxidation by treatment with MAP and antioxidants (Fig. 6).

**Figure 7:**
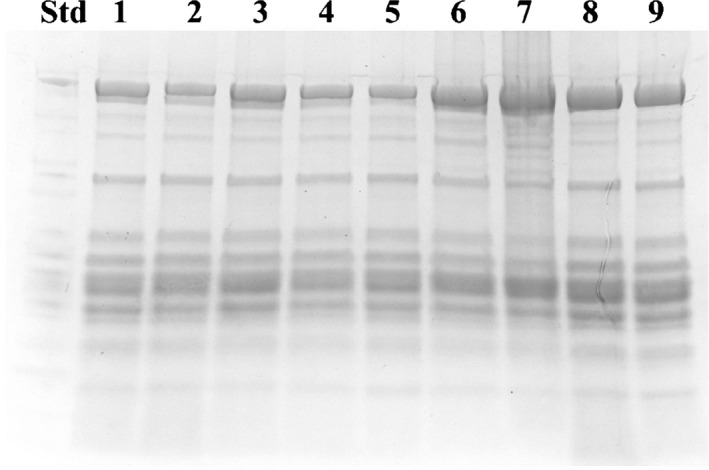
SDS‐PAGE profiles of total proteins extracted from *Coryphaena hippurus*, according to the method of preservation. Lane Std: wide range molecular weight markers; lane 1: whole fish (WH) at T zero; lane 2: WH at T3; lane 3: control group CO at T3; lane 4: modified atmosphere packaging MAP at T3; lane 5: MAP combined with natural antioxidant treatment MAP‐HAL (halophytes) at T3; lane 6: CO at T18; lane 7: MAP at T18; lane 8: MAP‐HAL (halophytes) at T18; lane 9: WH at T18.

The reduction of protein oxidation levels in MAP‐HAL fillets confirmed that HAL extract induced a significant antioxidant activity when coupled with MAP, also on protein fraction of the fillets and confirms our previous work, which demonstrated that MAP and antioxidants inhibited lipid oxidation in dolphinfish (Messina et al. 2015). Overall, the low levels of protein oxidation in both treated and untreated dolphinfish samples did not compromise the pattern and integrity of TP, as confirmed by SDS electrophoresis. No significant variations were observed between the electrophoretic patterns of the TP extracted from the samples, at the beginning and at the end of our study (Fig. 7).

### Correlations among sensorial, chemical, and biochemical parameters of quality

Sensorial aspects of quality in fish, including dolphinfish, are closely related to the chemical and biochemical composition of the fish and to their variations during storage (Du et al. 2000; Antoine et al. 2002b; Gómez‐Guillén et al. 2009). The temperature of preservation and the methods of processing contribute to preventing spoilage, preserving quality, and extending the shelf‐life of fish, as well as improving the overall quality (Mastromatteo et al. 2010; Masniyom 2011). In our study, the methods of processing effectively preserved the quality and shelf‐life of dolphinfish and improved some parameters related to sensorial, chemical, and biochemical quality in the MAP and MAP‐HAL groups compared to the untreated samples.

**Figure 8:**
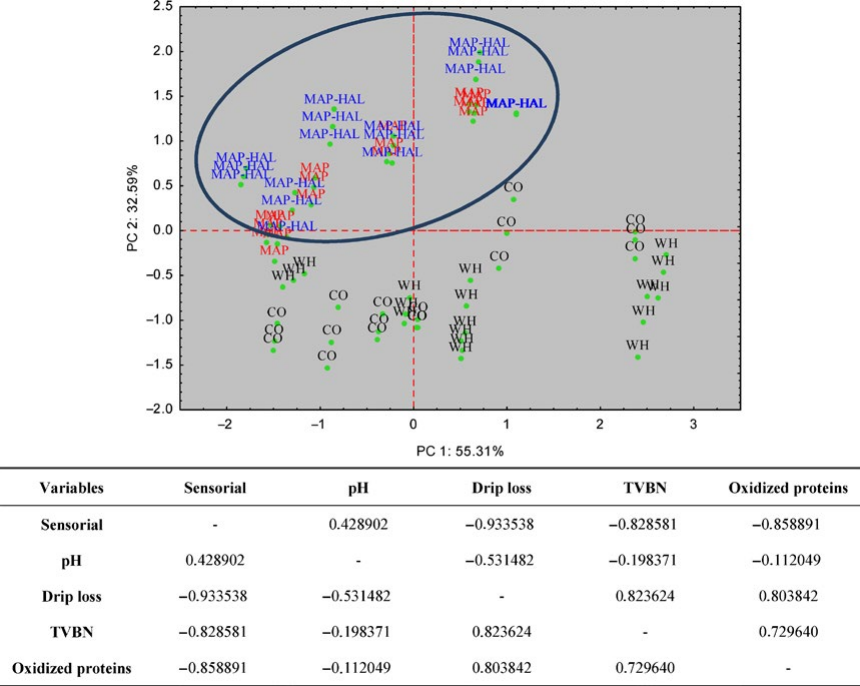
Principal component analysis, obtained from correlation of five components (sensorial analysis, pH, drip loss, TVBN, and oxidized proteins) and matrix of correlations among parameters. The 72 represented cases are mean of multiple determinations, collected on *Coryphaena hippurus* during the experiment according to the method of preservation: untreated control group CO and whole fish (black label), modified atmosphere packaging MAP (red label), MAP with natural antioxidant treatment MAP‐HAL (halophytes) (blue label).

PCA was applied to sensorial, chemical, and biochemical data obtained in this study (Fig. 8). Five variables (sensorial analysis, pH, drip loss, TVB‐N, and oxidized proteins) were considered for the analysis; PCA examined 72 cases that represented the means of data collected from WH fish and CO, MAP, and MAP‐HAL fillets, from day 3 to day 18. This analysis explained 87% of the variability in the original data (Fig. 8). The first principal component (PC1) explained 55.6% of the combined variance and the second component (PC2) explained 33.3% (Fig. 8). This representation indicates that the quality of untreated fish (WH fish and CO fillets) was different than the quality of MAP‐treated dolphinfish fillets (Fig. 8). By this representation, it is possible to appreciate that the points representing the quality of untreated fish, during the storage period, are dispersed within the graphic; on the contrary, the points representing the quality of MAP‐ and MAP‐HAL‐treated fillets are concentrated within the same area, independently from the time of preservation, indicating a more stable profile of quality in processed fillets than in untreated ones (Fig. 8). Finally, sensorial quality resulted strongly correlated to drip loss, TVB‐N level, and oxidized proteins, which confirms the effect of nitrogen and protein compounds on shelf‐life and perceived quality.

## Conclusions

Our results demonstrated that processing by filleting can ameliorate the quality evolution of dolphinfish during storage at −1°C. Overall, MAP, coupled with natural polyphenols from HAL, contributed to improved dolphinfish quality from sensorial, chemical, and biochemical points of view.

The biomarkers that are related to the quality of the proteins, such as TVB‐N and oxidized proteins, were lowest in samples processed by MAP and MAP‐HAL. The processing method that coupled MAP with natural antioxidants, resulted effective in extending the shelf‐life of dolphinfish fillets by almost 6 days, compared to WH fish. These findings support the application of this method of processing to increase both the shelf‐life and marketability of fishery species.

## Conflict of Interest

None declared.
